# Autoimmune signatures in neurodegenerative dementias: from peripheral immune activation to CNS pathology

**DOI:** 10.3389/fimmu.2026.1893167

**Published:** 2026-07-08

**Authors:** Xin Zhao, Guifeng Zhang, Zheng Wang, Dazheng Zhang, Zhangyong Xia, Chengju Huo

**Affiliations:** 1 Department of Neurology, Liaocheng People’s Hospital and Liaocheng Hospital Affiliated to Shandong First Medical University, Liaocheng, China; 2 Institute of Neuropathology, University Hospital RWTH Aachen, Aachen, Germany; 3 Department of Neurology, The Second People’s Hospital of Liaocheng, Liaocheng, Shandong, China

**Keywords:** adaptive immunity, autoantibodies, autoimmunity, neurodegenerative dementia, T cells

## Abstract

Neurodegenerative dementias, including Alzheimer’s disease, Parkinson’s disease dementia, dementia with Lewy bodies, and related tauopathies, are traditionally defined by protein aggregation, neuronal dysfunction, synaptic loss, and glial-mediated neuroinflammation. However, emerging evidence indicates that adaptive immunity may also contribute to disease heterogeneity and progression. These disorders should not be considered classical autoimmune diseases, but they may display autoimmune-like signatures, including neural antigen-specific T cell responses, clonal expansion of T cells in blood or cerebrospinal fluid, CNS infiltration of adaptive immune cells, and brain-targeting autoantibodies. Recent studies have linked α-synuclein-specific T cell reactivity to early Parkinson’s disease, identified clonally expanded CD8^+^ T cells in Alzheimer’s disease cerebrospinal fluid, and provided direct evidence of adaptive immune involvement in Lewy body dementia, including altered peripheral immunophenotypes and CD4^+^ T cell-associated neurodegenerative mechanisms. Experimental tauopathy models further show that microglia-mediated T cell infiltration can drive neurodegeneration. Humoral autoreactivity and progression-associated immune changes further suggest that adaptive immune profiles may help define biologically distinct dementia subgroups. In this mini review, we summarize evidence connecting peripheral immune activation, intrathecal adaptive immune remodeling, and CNS pathology in neurodegenerative dementias. We also discuss how longitudinal blood–CSF profiling, single-cell/TCR/BCR sequencing, autoantibody profiling, and mechanistic validation may clarify whether these immune signatures are pathogenic, compensatory, or bystander responses.

## Highlights

- Neurodegenerative dementias may exhibit autoimmune-like adaptive immune signatures without being classical autoimmune diseases.
- Peripheral antigen-specific T cell responses, CSF T cell clonality, CNS infiltration, and brain-targeting autoantibodies may link systemic immunity to neuropathology.
- Longitudinal blood–CSF immune profiling and mechanistic studies are needed to distinguish pathogenic, compensatory, and bystander immune responses.

## 1 Introduction

Neurodegenerative dementias, including Alzheimer’s disease (AD), Parkinson’s disease dementia (PDD), dementia with Lewy bodies, and related tauopathies, have traditionally been understood through the lens of protein aggregation, neuronal dysfunction, synaptic loss, and glial-mediated neuroinflammation (1–3). In AD, amyloid-β (Aβ) deposition and tau pathology are central pathological hallmarks, whereas α-synuclein aggregation is a defining feature of Parkinson’s disease (PD) and Lewy body-related dementias (4, 5). Although much of the adaptive immune literature has been generated from AD and classical PD cohorts, emerging studies have begun to provide disease-relevant evidence for Lewy body dementia, including DLB and PDD (6, 7). Peripheral immunophenotyping studies have shown altered T cell and B cell-related immune profiles in DLB compared with AD and controls, while post-mortem and experimental synucleinopathy studies have reported T cell infiltration in Lewy body disease and α-synuclein models (8). In addition, single-cell and CSF-based analyses in Lewy body dementia suggest that adaptive immune remodeling may occur at both systemic and CNS-associated immune interfaces (9). Therefore, in this review, PD-derived α-synuclein immune findings are discussed as part of a broader α-synucleinopathy framework, but DLB/PDD-specific evidence is highlighted where available. These disease-associated proteinopathies are accompanied by progressive neuronal injury, impaired proteostasis, mitochondrial dysfunction, vascular alterations, and activation of innate immune cells such as microglia and astrocytes. For many years, neuroinflammation in dementia was therefore largely interpreted as a CNS-intrinsic process dominated by resident glial cells.

However, this view has become increasingly incomplete. Accumulating evidence indicates that the adaptive immune system may also participate in the initiation, progression, or modulation of neurodegenerative dementias. Peripheral T cells, B cells, clonally expanded lymphocyte populations, cerebrospinal fluid (CSF)-resident immune cells, and brain-targeting autoantibodies have all been detected in subsets of patients with neurodegenerative disorders (10–13). These findings suggest that neurodegeneration may not be fully separated from systemic immune dysregulation. Instead, peripheral immune activation and CNS pathology may interact through dynamic immune trafficking, antigen recognition, blood–brain barrier disruption, meningeal and CSF immune surveillance, and glia-mediated antigen presentation.

Importantly, neurodegenerative dementias should not be simply reclassified as classical autoimmune diseases. Unlike prototypical autoimmune disorders, such as multiple sclerosis or autoimmune encephalitis, AD and PD do not usually present with a single dominant pathogenic autoantigen, robust inflammatory demyelination, or rapid steroid-responsive disease courses (14–16). Nevertheless, these disorders may exhibit autoimmune-like signatures, defined here as adaptive immune features that resemble components of autoimmunity without necessarily fulfilling the criteria for a classical autoimmune disease. Such signatures may include neural antigen-specific T cell responses, clonal expansion of T cells in blood or CSF, CNS infiltration of cytotoxic or antigen-experienced lymphocytes, B cell activation, and brain-reactive autoantibodies.

Several recent experimental studies have brought this concept into sharper focus. In AD, Gate et al. reported clonally expanded CD8^+^ T cells in the CSF, suggesting that antigen-experienced adaptive immune cells may patrol the intrathecal compartment in association with disease pathology (17). This finding supports the idea that the CSF may serve as an immunological interface between peripheral immunity and CNS neurodegeneration. In PD, Lindestam Arlehamn et al. showed that α-synuclein-specific T cell reactivity is associated with preclinical and early disease stages, indicating that adaptive immune recognition of disease-associated neural antigens may arise before or around the time of clinical diagnosis (18). Together, these studies suggest that adaptive immune responses may not merely represent late bystander inflammation but could be temporally and biologically linked to early disease processes. The concept of autoimmune signatures is particularly valuable because it allows a more nuanced interpretation of immune findings in neurodegenerative dementias. Rather than asking whether AD or PD is an autoimmune disease, a more productive question is whether specific patient subsets display adaptive immune patterns that influence disease trajectory, reflect CNS pathology, or identify therapeutic windows. For example, neural antigen-reactive T cells may contribute to pathology in some contexts but may also represent compensatory immune surveillance or age-related autoreactivity in others. Similarly, brain-targeting autoantibodies may be pathogenic, secondary to neuronal damage, or useful as biomarkers of blood–brain barrier dysfunction and immune exposure to CNS antigens.

In this mini review, we discuss emerging evidence for autoimmune-like adaptive immune signatures in neurodegenerative dementias, with a focus on three interconnected levels: peripheral immune activation, CSF/CNS adaptive immune remodeling, and humoral autoreactivity. We highlight how neural antigen-specific T cell responses, T cell clonal expansion, CNS immune infiltration, and brain-targeting autoantibodies may connect systemic immune dysregulation to neurodegenerative pathology. Finally, we consider how these signatures may inform disease staging, biomarker discovery, and future immune-modulatory strategies for selected patient subgroups.

## 2 Peripheral autoimmune-like activation in early neurodegeneration

A central question in the study of autoimmune-like signatures in neurodegenerative dementias is whether adaptive immune activation occurs only as a late consequence of neuronal injury, or whether it may emerge during early disease stages and contribute to subsequent pathological progression. Traditionally, peripheral immune alterations in Alzheimer’s disease (AD), Parkinson’s disease (PD), and related dementias were often interpreted as secondary responses to chronic neurodegeneration, systemic aging, or nonspecific inflammation. However, recent evidence suggests that adaptive immune changes can be detected before extensive clinical deterioration and may be linked to disease-associated neural antigens or early CNS pathology. This raises the possibility that peripheral autoimmune-like activation may represent an early immunological signal in selected neurodegenerative conditions.

### 2.1 Parkinson’s disease: α-synuclein-specific T cell reactivity

PD provides one of the clearest examples of peripheral antigen-specific adaptive immune activation in neurodegeneration. α-Synuclein is a major pathological protein in PD and Lewy body-related dementias, and its aggregation is central to dopaminergic neuronal degeneration and disease progression (19–21). Beyond its role as a misfolded neuronal protein, α-synuclein may also function as an immune-recognized antigen. In a key study, Lindestam Arlehamn et al. demonstrated that α-synuclein-specific T cell reactivity is associated with preclinical and early PD (18). This finding is important because it links a disease-defining neural antigen to peripheral T cell responses at a stage when neurodegenerative processes may already be underway but before advanced disease has developed.

The implication of this study is not that PD should be considered a classical autoimmune disease. Rather, it suggests that disease-associated neural antigens can be recognized by the peripheral adaptive immune system. Such recognition may arise through several non-mutually exclusive mechanisms, including antigen release from injured neurons, altered antigen processing, peripheral presentation of CNS-derived proteins, or changes in immune tolerance during aging and chronic inflammation. The temporal association with early PD is particularly relevant, because it suggests that α-synuclein-specific T cell responses may not simply reflect end-stage tissue destruction. Instead, they may represent an early autoimmune-like signature that accompanies or potentially modulates disease development (22–25). Nevertheless, caution is required when interpreting α-synuclein-specific T cell reactivity. Antigen recognition does not automatically prove pathogenic autoimmunity. These T cells may contribute to neuronal injury, but they may also reflect immune surveillance, clearance responses against abnormal protein aggregates, or bystander activation in an inflammatory environment. Therefore, the key question is not only whether α-synuclein-reactive T cells exist, but also what functional phenotype they adopt, whether they produce inflammatory or cytotoxic mediators, whether they traffic to CNS-associated compartments, and whether their frequency or activity predicts disease progression.

### 2.2 Lewy body dementias: DLB/PDD-specific adaptive immune evidence

Lewy body dementias, including dementia with Lewy bodies and Parkinson’s disease dementia, provide an important test case for determining whether α-synuclein-associated adaptive immune signatures extend beyond classical PD (6). Although direct DLB/PDD immune datasets remain fewer than those available for AD or PD, several studies support the relevance of adaptive immune remodeling in Lewy body dementia (8, 9). Peripheral immunophenotyping has revealed altered immune profiles in DLB, including changes in T cell subsets, B cell activation, and pro-inflammatory cytokine patterns (8). These findings suggest that DLB may have a partially distinct peripheral immune signature rather than simply mirroring AD or PD (8, 26). At the CNS level, studies of Lewy body disease and α-synucleinopathy models have reported T cell infiltration near affected brain regions and have implicated CD4^+^ T cells in neurodegenerative mechanisms (9, 27). Single-cell and CSF-based analyses further indicate that adaptive immune cells in Lewy body dementia may acquire disease-associated trafficking or activation features. Together, these findings provide empirical support for including DLB/PDD within the scope of autoimmune-like signatures, while also emphasizing that DLB-specific longitudinal blood–CSF studies remain limited. Therefore, conclusions derived from classical PD cohorts should be interpreted cautiously and should not be automatically generalized to DLB without disease-specific validation.

### 2.3 Alzheimer’s disease: peripheral T cell alterations linked to amyloid pathology

In AD, the relationship between peripheral adaptive immunity and early CNS pathology is also gaining attention. Aβ deposition is one of the earliest detectable pathological changes in AD, often preceding overt dementia by many years. If peripheral immune alterations are already associated with early amyloid accumulation, this would support the concept that systemic immune remodeling may reflect, respond to, or influence preclinical neurodegenerative pathology.

Gericke et al. provided evidence supporting this possibility by showing that early β-amyloid accumulation in the brain is associated with peripheral T cell alterations (28). This study is important for the present review because it connects an early CNS pathological event with measurable changes in peripheral adaptive immunity. Rather than focusing only on advanced dementia, it places immune alterations within the early biological continuum of AD. Such findings suggest that peripheral immune profiling may provide information about disease stage, amyloid burden, or immune–brain communication before severe cognitive decline occurs. The biological interpretation of these peripheral T cell changes remains complex. Peripheral T cell alterations may reflect systemic immune aging, chronic inflammatory priming, altered antigen exposure, or communication between the CNS and peripheral immune compartments through CSF drainage, meningeal immunity, lymphatic pathways, or blood–brain barrier dysfunction. In this context, peripheral immune signatures should be viewed as potential indicators of CNS pathological states rather than direct proof of autoimmune causality. However, their association with early amyloid pathology supports the broader concept that neurodegenerative diseases are not fully isolated within the brain. Instead, disease-associated proteinopathy may be accompanied by systemic adaptive immune remodeling.

### 2.4 Conflicting evidence: antigen reactivity is not always disease-specific

Although these studies support the relevance of peripheral autoimmune-like activation, the field must avoid overinterpreting antigen reactivity as disease-specific autoimmunity. This is particularly important in aging-related neurodegenerative diseases, where low-level autoreactive T cells may exist in healthy individuals and where immune senescence can alter T cell repertoires independently of dementia pathology. Dhanwani et al. addressed this issue by examining T cell responses to neural autoantigens in AD patients and age-matched healthy controls (29). Their study found that T cell responses to neural antigens were broadly similar between AD patients and controls, suggesting that antigen reactivity alone may not be sufficient to distinguish disease-associated immune activation from age-related autoreactivity. This finding provides an important counterbalance to more disease-positive studies. It indicates that autoimmune-like signatures in neurodegenerative dementias should not be defined simply by the presence or absence of T cell reactivity to neural antigens. Instead, their interpretation requires attention to disease stage, antigen specificity, HLA context, T cell phenotype, cytokine profile, clonal expansion, tissue compartment, and longitudinal dynamics. For example, a neural antigen-reactive T cell population may have different implications depending on whether it is rare or expanded, naïve or memory-like, regulatory or cytotoxic, confined to blood or enriched in CSF, stable over time or associated with clinical progression.

**Figure 1:**
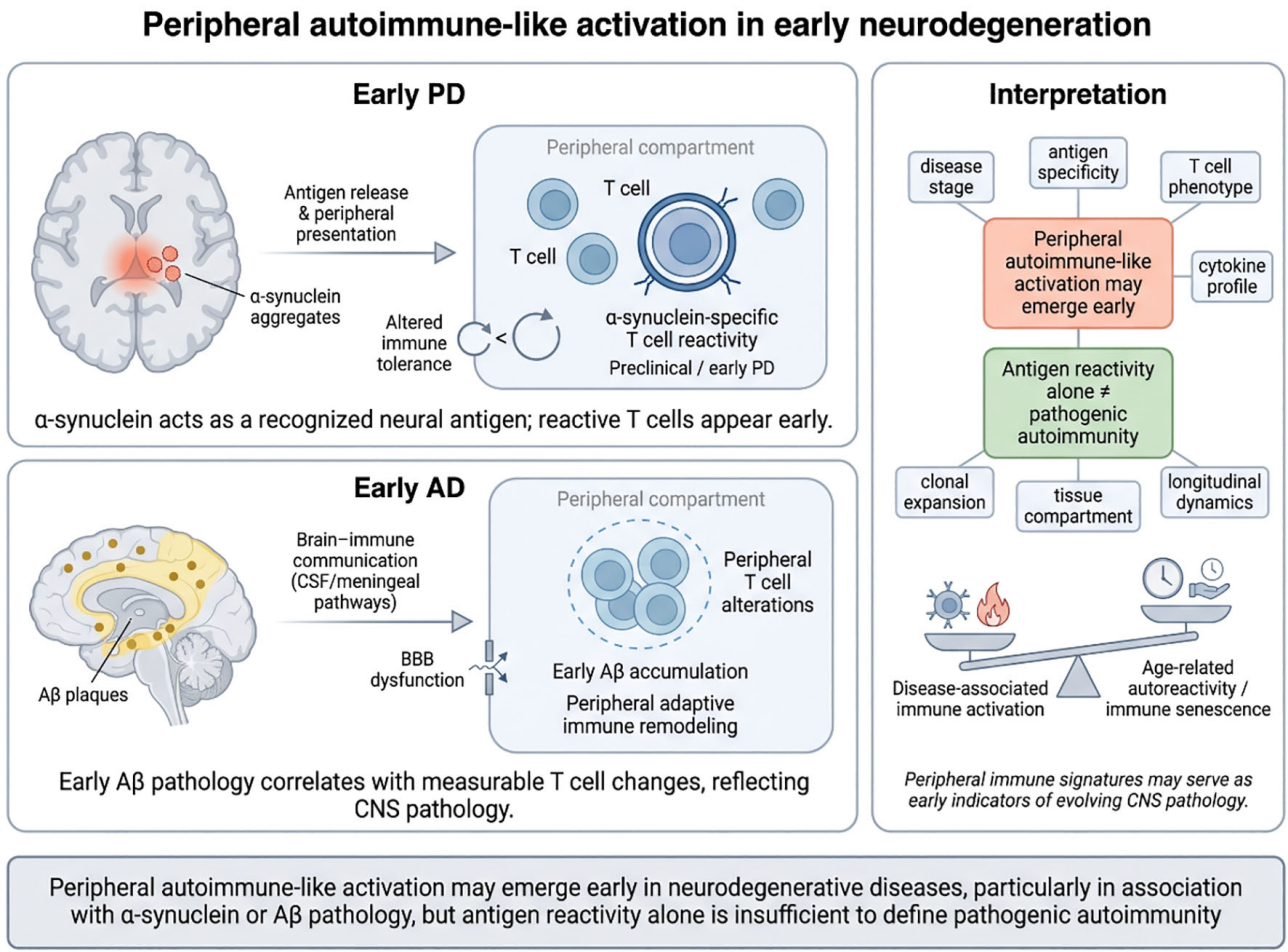
Peripheral autoimmune-like activation in early neurodegeneration. Disease-associated neural proteins, including α-synuclein in Parkinson’s disease and amyloid-β in Alzheimer’s disease, may be linked to early peripheral T cell alterations. These immune changes may reflect antigen recognition, peripheral immune remodeling, or brain–immune communication, but antigen reactivity alone is insufficient to establish pathogenic autoimmunity.

Taken together, current evidence suggests that peripheral autoimmune-like activation may emerge early in neurodegenerative diseases, particularly when linked to disease-associated proteins such as α-synuclein or Aβ. However, antigen reactivity alone is insufficient to define pathogenic autoimmunity. A more precise framework is needed—one that integrates antigen specificity with immune cell function, clonal architecture, disease stage, and CNS pathological context. In this sense, peripheral adaptive immune changes may serve less as proof that neurodegenerative dementias are autoimmune diseases, and more as early immune signatures that help reveal how systemic immunity interacts with evolving CNS pathology. **Figure 1** illustrates how early PD- and AD-associated proteinopathies may be accompanied by peripheral autoimmune-like adaptive immune activation. It also emphasizes that neural antigen reactivity should be interpreted in the context of disease stage, T cell phenotype, clonal expansion, and CNS pathological status rather than being equated directly with pathogenic autoimmunity.

## 3 CSF and CNS adaptive immune signatures: the bridge to neuropathology

Although peripheral immune alterations provide important evidence for systemic adaptive immune involvement in neurodegenerative diseases, they do not by themselves explain how immune responses become connected to tissue-level neuropathology. A critical question is therefore how peripheral autoimmune-like signals communicate with, enter, or reflect the CNS environment. The cerebrospinal fluid (CSF), meninges, perivascular spaces, and brain parenchyma represent key anatomical and immunological interfaces between systemic immunity and neurodegenerative pathology. In this context, CSF and CNS adaptive immune signatures may serve as an intermediate layer linking peripheral immune activation to local neuronal injury, glial activation, and proteinopathy-driven neurodegeneration.

### 3.1 Meningeal lymphatics and border-associated macrophages as anatomical immune bridges

An important anatomical question is where CNS-derived antigens are sampled and where peripheral-to-central adaptive immune communication may physically occur. Meningeal lymphatic vessels provide a key drainage route through which cerebrospinal fluid, interstitial solutes, immune cells, and CNS-derived macromolecules can reach deep cervical lymph nodes (30–32). In neurodegenerative dementias, this pathway may be particularly relevant because disease-associated aggregates, such as amyloid-β and potentially other misfolded proteins, can be cleared or exposed through meningeal and perivascular drainage routes (33, 34). Impaired meningeal lymphatic function during aging or Alzheimer’s disease may therefore reduce protein aggregate clearance, alter microglial responses, and reshape immune surveillance (33, 34). In parallel, border-associated macrophages located at meningeal, perivascular, and choroid plexus interfaces are strategically positioned to sample CSF- or vessel-associated antigens, respond to vascular and inflammatory cues, and participate in antigen presentation to adaptive immune cells (35, 36). Together, meningeal lymphatics and border-associated macrophages may form an anatomical bridge between CNS proteinopathy and peripheral adaptive immune priming. This framework helps explain how CNS-derived antigens could become visible to circulating lymphocytes and why CSF immune signatures may reflect not only passive leakage from blood, but active immune surveillance at CNS border compartments. However, direct evidence linking these border immune structures to disease-specific TCR or BCR clones in AD, PD, DLB/PDD, and tauopathies remains limited and should be prioritized in future longitudinal studies.

### 3.2 Clonally expanded CD8^+^ T cells in AD CSF

One of the strongest pieces of evidence supporting intrathecal adaptive immune involvement in Alzheimer’s disease (AD) comes from the study by Gate et al., who identified clonally expanded CD8^+^ T cells in the CSF of patients with AD (17). This finding was important because it shifted attention from general systemic inflammation toward compartment-specific adaptive immune remodeling. The presence of clonally expanded, antigen-experienced CD8^+^ T cells suggests that the CSF is not merely a passive fluid compartment but may function as an immunological surveillance space in which adaptive immune cells interact with CNS-derived antigens or inflammatory cues (37).

The concept of clonal expansion is particularly relevant to autoimmune-like signatures. In adaptive immunity, clonal expansion usually reflects antigen-driven selection or repeated stimulation. Therefore, expanded CD8^+^ T cell clones in AD CSF raise the possibility that specific antigens, potentially derived from infected cells, damaged neurons, glial cells, or disease-associated CNS proteins, may shape the local immune repertoire (38–41). However, the precise antigenic targets of these T cells remain unclear. This uncertainty is important: clonal expansion indicates immune selection, but it does not automatically prove that these cells recognize Aβ, tau, or other AD-related autoantigens. They may also respond to viral antigens, age-associated inflammatory signals, or tissue damage-associated cues. Nevertheless, the study provides a conceptual bridge between peripheral immune activation and CNS pathology. If antigen-experienced CD8^+^ T cells patrol the CSF in AD, then CSF immune profiling may capture adaptive immune events that are closer to CNS disease biology than peripheral blood alone. This has major implications for biomarker development. Blood-based immune changes may be influenced by systemic aging, infection history, metabolic status, and comorbidities, whereas CSF immune signatures may more directly reflect CNS immune surveillance, blood–brain barrier alterations, and local neuroinflammatory states. Thus, CSF T cell clonality may represent an important autoimmune-like signature linking systemic immunity with neurodegenerative pathology.

### 3.3 Single-cell and TCR evidence in PD

Similar evidence for adaptive immune remodeling has also emerged in Parkinson’s disease (PD). Wang et al. used single-cell transcriptome and T cell receptor (TCR) profiling to reveal activated and expanded T cell populations in patients with PD (42). This study is particularly valuable because it combined cellular phenotyping with TCR-level information, allowing the authors to move beyond simple immune cell abundance and examine the clonal and functional architecture of T cells.

The identification of activated and expanded T cell populations in PD supports the idea that adaptive immunity is not simply a nonspecific inflammatory background. Instead, T cells in PD may undergo disease-associated remodeling, including activation, differentiation, and clonal expansion. TCR profiling provides an additional layer of evidence because expanded TCR clonotypes may suggest antigen-driven or repeated immune stimulation. When considered together with earlier evidence of α-synuclein-specific T cell reactivity, these findings strengthen the possibility that PD involves an adaptive immune component linked to disease-associated antigens or CNS-derived signals. Single-cell approaches are especially useful in this field because neurodegenerative diseases are immunologically heterogeneous. Bulk immune profiling may miss rare but biologically important T cell populations, such as cytotoxic CD4^+^ T cells, terminal effector CD8^+^ T cells, tissue-homing T cells, or exhausted antigen-experienced clones (43–47). By contrast, single-cell transcriptomics combined with TCR sequencing can identify functional states, clonal relationships, and potential disease-associated immune trajectories. In PD, such approaches help define whether adaptive immune changes are dominated by cytotoxicity, chronic activation, memory differentiation, or regulatory dysfunction.

However, as in AD, interpretation requires caution. T cell activation and clonal expansion do not necessarily prove direct neurotoxicity. These changes may reflect immune responses to peripheral inflammation, infections, altered gut immunity, systemic aging, or CNS antigen exposure secondary to neuronal injury. Therefore, the major value of the Wang et al. study is not that it definitively proves autoimmune causality in PD, but that it provides a high-resolution map of adaptive immune remodeling that can be integrated with antigen specificity, CSF findings, disease stage, and clinical progression.

### 3.4 Microglia-mediated T cell infiltration in tauopathy

While CSF and blood studies reveal adaptive immune signatures associated with neurodegenerative diseases, experimental models are needed to test whether immune cells can actively contribute to CNS pathology. In this regard, the study by Chen et al. provides one of the most compelling mechanistic links between CNS proteinopathy, microglial activation, T cell infiltration, and neurodegeneration (48). Using tauopathy models, the authors showed that microglia-mediated T cell infiltration can drive neurodegenerative processes. Importantly, depletion of microglia or T cells attenuated neurodegeneration, providing evidence that this immune axis is not merely correlative but may contribute causally to tissue damage. This study is highly relevant to the concept of autoimmune-like signatures because it places adaptive immune cells within the diseased CNS microenvironment. Tau pathology may activate microglia, and activated microglia may in turn produce inflammatory mediators, chemokines, or antigen-presenting signals that promote T cell recruitment or retention (49–52). Once present in the CNS, T cells may amplify neuroinflammation through cytokine production, cytotoxic mechanisms, or interactions with glial and neuronal cells. In this model, neurodegeneration is not driven solely by misfolded tau or glial activation, but by a pathological circuit in which proteinopathy, innate immunity, and adaptive immunity reinforce one another.

The microglia–T cell axis also provides an explanation for why peripheral immune activation does not always translate into CNS pathology. Peripheral T cells may require local CNS cues, including chemokine gradients, antigen presentation, blood–brain barrier disruption, or inflammatory microenvironments, before they can exert tissue-level effects (53–56). At the molecular level, chemokine-guided trafficking provides a plausible mechanism by which peripheral T cells are recruited to CSF and CNS-associated compartments (17, 48). Among these pathways, the CXCL9/CXCL10–CXCR3 axis is particularly relevant (57). Activated microglia, astrocytes, endothelial cells, and other inflamed CNS-resident cells can produce interferon-inducible chemokines such as CXCL9 and CXCL10, which bind CXCR3 expressed on activated, effector, or clonally expanded T cells (12, 57). This ligand–receptor interaction may promote T cell adhesion, directional migration, and retention within inflamed CNS niches (12, 58). In Alzheimer’s disease models, CXCL10–CXCR3 signaling has been shown to regulate CD8^+^ T cell infiltration and T cell-mediated neuronal injury, while CXCR3 deficiency reduces amyloid pathology and behavioral deficits in APP/PS1 mice (12). These findings suggest that chemokine axes do not merely accompany neuroinflammation but may actively orchestrate central–peripheral immune cross-talk. However, whether the same CXCL9/CXCL10–CXCR3 mechanism operates uniformly across AD, PD, DLB/PDD, and tauopathies remains unresolved and requires disease-specific validation. Thus, CNS context determines whether adaptive immune cells remain peripheral bystanders, CSF surveillance cells, or active participants in neurodegeneration. This is particularly important for tauopathies, where regional neurodegeneration, microglial activation, and T cell infiltration may converge spatially and temporally.

Taken together, these studies support a layered model of adaptive immune involvement in neurodegenerative diseases. Peripheral immune activation may provide the first detectable autoimmune-like signal; CSF immune surveillance may represent a transitional compartment where antigen-experienced and clonally expanded T cells can be detected; and CNS infiltration may occur when local glial activation and proteinopathy create permissive conditions for adaptive immune recruitment. In this framework, CSF and CNS adaptive immune signatures are not isolated observations, but part of a continuum connecting systemic immune remodeling to tissue-level neurodegeneration.

**Figure 2:**
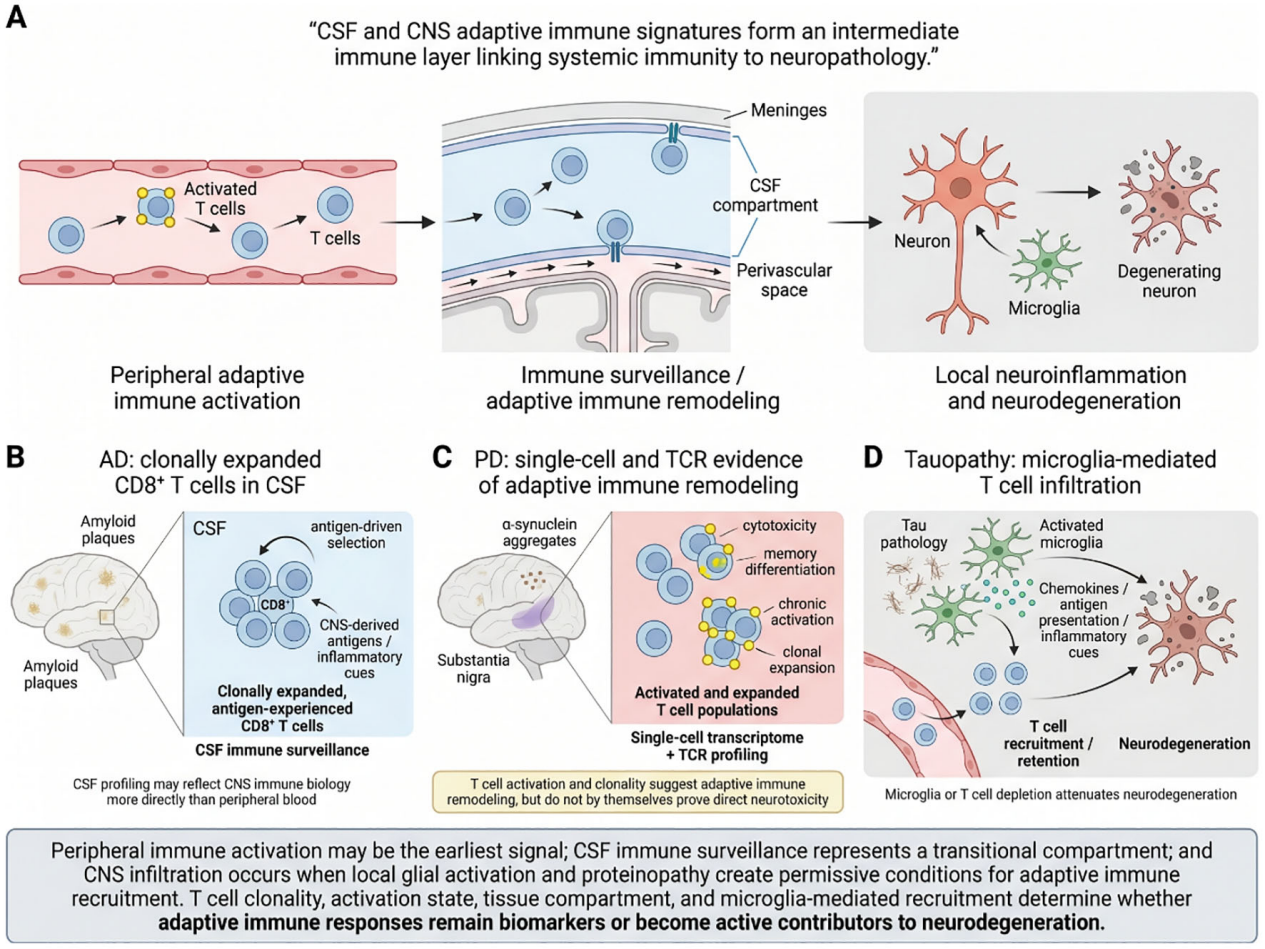
CSF and CNS adaptive immune signatures linking peripheral activation to neuropathology. Peripheral adaptive immune activation may extend into CSF and CNS border compartments, where antigen-experienced or clonally expanded T cells reflect immune surveillance and adaptive immune remodeling. In tauopathy, local microglial activation may further promote T cell recruitment into brain parenchyma, amplifying neuroinflammation and neurodegeneration.

Overall, CSF and CNS adaptive immune signatures may represent an intermediate immune layer linking peripheral activation to neuropathological injury. Studies in AD, PD, and tauopathy suggest that T cell clonality, activation state, tissue compartmentalization, and microglia-mediated recruitment are key variables that determine whether adaptive immune responses remain biomarkers of disease or become active contributors to neurodegeneration. **Figure 2** illustrates a layered immune continuum from peripheral adaptive immune activation to CSF immune surveillance and CNS T cell infiltration. It highlights how T cell clonality, activation state, tissue compartmentalization, and microglia-mediated recruitment may determine whether adaptive immune responses remain biomarkers or actively contribute to neurodegeneration.

## 4 Humoral and progression-associated autoimmune signatures

Compared with T cell-mediated mechanisms, humoral autoimmune-like signatures in neurodegenerative dementias have received less attention. However, B cells, immunoglobulins, and brain-reactive autoantibodies may represent an additional layer of adaptive immune involvement. In classical autoimmune neurological diseases, pathogenic antibodies can directly bind neuronal or glial targets, alter receptor function, activate complement, or promote inflammatory tissue injury. In neurodegenerative dementias, the situation is likely more complex. Brain-targeting antibodies may contribute to pathology in selected patients, but they may also arise secondary to blood–brain barrier (BBB) disruption, neuronal damage, impaired immune tolerance, or age-associated immune dysregulation. Therefore, humoral autoreactivity should be interpreted as an autoimmune-like signature rather than immediate proof of antibody-mediated disease.

### 4.1 Brain-targeting autoantibodies in dementia

A recent study by Staabs et al. provided direct evidence that brain-targeting autoantibodies can be detected in patients with dementia (59). The authors examined IgG and IgA autoantibodies directed against brain antigens in dementia patients and controls, identifying a higher frequency of brain-reactive autoantibodies in the dementia group. This finding is relevant because it expands the concept of autoimmune signatures beyond T cell reactivity and clonal expansion. It suggests that humoral autoreactivity may also accompany neurodegenerative cognitive disorders and may help define a subgroup of patients with enhanced immune recognition of CNS-related antigens.

The presence of brain-targeting IgG or IgA antibodies raises several mechanistic possibilities (60–63). First, these antibodies could be directly pathogenic if they bind functionally important neuronal, synaptic, glial, or vascular targets and gain access to the CNS. In this scenario, autoantibodies might amplify synaptic dysfunction, glial activation, complement-mediated injury, or neuronal stress. Second, these antibodies may represent a consequence of BBB disruption (64–66). Neurodegenerative diseases are frequently associated with vascular dysfunction, barrier leakage, and altered immune surveillance, all of which may expose CNS antigens to the peripheral immune system and allow circulating antibodies to access the brain. Third, humoral autoreactivity may arise after neuronal injury, when intracellular or normally sequestered brain antigens are released and processed by antigen-presenting cells. In this case, autoantibodies may be secondary markers of tissue damage rather than primary drivers of neurodegeneration. This distinction is essential for the interpretation of autoantibody findings in dementia. In autoimmune encephalitis, the relationship between autoantibodies and neurological symptoms is often clearer, and immunotherapy can produce substantial clinical improvement. Recent clinical cohort studies further highlight why autoimmune encephalitis should be considered as an important clinical control context when discussing autoimmune-like signatures in dementia (67, 68). Autoimmune encephalitis can resemble neurodegenerative dementia syndromes, especially in middle-aged or older patients presenting with prominent cognitive decline, subtle seizures, or atypical ancillary findings (67). In a nationwide observational cohort, a substantial proportion of older autoimmune encephalitis patients fulfilled dementia criteria, and neurodegenerative dementia was initially suspected in many cases (67). Importantly, some patients lacked typical inflammatory changes on MRI or CSF examination, indicating that the absence of overt inflammation does not fully exclude an autoimmune mimic (67, 69). A more recent memory-clinic cohort of patients with presumed neurodegenerative dementia also showed that neuronal antibodies associated with autoimmune encephalitis can be detected in a small but clinically relevant subgroup when stringent multi-assay confirmation is applied (68). These findings provide a practical clinical rationale for targeted autoantibody screening and multi-layered immune profiling in patients with atypical, rapidly progressive, fluctuating, or otherwise diagnostically uncertain dementia presentations. However, antibody testing should be interpreted cautiously and in clinical context, because false-positive serum results and overdiagnosis of autoimmune encephalitis may lead to inappropriate immunotherapy. By contrast, in slowly progressive neurodegenerative dementias, brain-reactive antibodies may not have the same pathogenic meaning as antibodies identified in classical autoimmune encephalitis (59). Some antibodies may be epiphenomena of chronic tissue injury, BBB disruption, or age-associated immune dysregulation, whereas others may identify treatable or partially immune-responsive subgroups. Future studies will therefore need to determine whether antibody-positive patients differ in disease course, BBB integrity, neuroinflammatory biomarkers, imaging patterns, or response to immunomodulatory therapy. Functional validation will also be necessary to test whether these antibodies alter neuronal activity, glial function, complement activation, or synaptic integrity.

### 4.2 Adaptive immune changes associated with AD progression

In addition to humoral autoreactivity, adaptive immune changes may also correlate with disease progression. This is important because autoimmune-like signatures may be more useful if they can help stratify patients by disease stage, trajectory, or biological subtype. van Olst et al. reported that adaptive immune changes are associated with clinical progression of Alzheimer’s disease (70). Their findings support the idea that peripheral immune remodeling is not static but may evolve across the clinical course of AD and relate to markers of neurodegeneration, inflammation, or cognitive decline. This progression-associated perspective is valuable for several reasons. First, it suggests that adaptive immune profiles may serve as dynamic biomarkers rather than fixed diagnostic labels (26, 71–73). For example, changes in memory T cells, B cell subsets, or other adaptive immune populations may reflect transitions from preclinical disease to mild cognitive impairment and dementia (74–77). Second, progression-associated immune signatures may help distinguish patients with stronger immune involvement from those whose disease is dominated by other mechanisms, such as metabolic dysfunction, vascular pathology, or primary protein aggregation. Third, immune changes linked to disease progression may provide clues about therapeutic timing. If adaptive immune activation is more prominent at specific disease stages, immunomodulatory approaches may need to be stage-specific rather than broadly applied to all patients with dementia.

The integration of humoral and progression-associated signatures also highlights the need for multi-layered immune profiling. Autoantibodies alone may be insufficient to define a pathogenic immune subtype. Similarly, changes in peripheral immune cell populations may be difficult to interpret without information on antibody repertoires, CSF biomarkers, BBB function, T cell clonality, and CNS pathology. A more informative approach would combine blood and CSF immune profiling with Aβ, tau, neurofilament light chain, inflammatory markers, and clinical progression data. Such integrated analyses could clarify whether humoral autoreactivity and adaptive immune remodeling are causes, consequences, or modifiers of neurodegeneration.

Overall, humoral autoreactivity and progression-associated adaptive immune profiles may help define immune subtypes of dementia, although their causal relevance remains unresolved. Rather than implying that all neurodegenerative dementias are antibody-mediated or autoimmune diseases, these findings suggest that selected patients may display measurable adaptive immune signatures with potential biomarker and therapeutic relevance. Future longitudinal studies will be essential to determine whether these signatures precede pathology, parallel disease progression, or emerge as secondary consequences of CNS injury.

## 5 Conclusion and future perspectives

Neurodegenerative dementias should not be regarded as classical autoimmune diseases. Unlike prototypical autoimmune neurological disorders, such as multiple sclerosis or autoimmune encephalitis, Alzheimer’s disease, Parkinson’s disease dementia, dementia with Lewy bodies, and related tauopathies are primarily defined by progressive proteinopathy, neuronal dysfunction, synaptic loss, and glial-mediated neuroinflammation. They generally lack a single dominant pathogenic autoantigen, a uniformly inflammatory clinical course, or consistent responsiveness to immunosuppressive therapy. Therefore, it would be overly simplistic to classify these disorders as autoimmune diseases in the conventional sense.

Nevertheless, accumulating evidence indicates that neurodegenerative dementias can display autoimmune-like adaptive immune signatures. These signatures may include peripheral T cell reactivity against disease-associated neural antigens, clonal expansion of T cells in blood or cerebrospinal fluid, recruitment of adaptive immune cells into CNS-associated compartments, microglia-mediated T cell infiltration in the context of tauopathy, and brain-targeting autoantibodies. Together, these observations suggest that adaptive immunity may participate in neurodegenerative disease biology at multiple levels, from systemic immune activation to intrathecal immune surveillance and tissue-level neuroinflammatory amplification.

A central challenge for the field is to determine the biological meaning of these immune signatures. Some adaptive immune responses may be pathogenic, directly contributing to neuronal injury through cytotoxicity, inflammatory cytokine production, complement activation, or antibody-mediated mechanisms. Others may be compensatory, reflecting attempts to clear abnormal protein aggregates, maintain immune surveillance, or limit tissue damage. Still others may be bystander responses secondary to aging, chronic inflammation, blood–brain barrier disruption, or release of CNS antigens after neuronal injury. Distinguishing among these possibilities is essential before autoimmune-like signatures can be translated into therapeutic strategies.

Future research should therefore move beyond cross-sectional immune profiling toward longitudinal and mechanistically integrated study designs. Paired blood–CSF cohorts will be particularly important for determining whether peripheral immune alterations precede, parallel, or follow CNS pathological changes. Single-cell transcriptomics, T cell receptor and B cell receptor sequencing, autoantibody profiling, and high-dimensional proteomic or cytokine analyses should be integrated with established biomarkers such as amyloid-β, tau, neurofilament light chain, neuroimaging markers, and cognitive trajectories. Such approaches may help identify immune subtypes of dementia and clarify whether specific adaptive immune signatures are associated with disease onset, progression, or treatment response.

Experimental validation will also be essential. Animal models, humanized immune models, organoid-based systems, and co-culture platforms may help test whether antigen-specific T cells, clonally expanded lymphocytes, brain-reactive antibodies, or microglia–T cell interactions directly contribute to neurodegeneration. These models should also determine when immune modulation is beneficial or harmful, since broad immunosuppression may disrupt protective immune surveillance, tissue repair, and host defense. Therapeutically, the most promising direction is unlikely to be generalized immune inhibition, but rather precise modulation of disease-relevant immune circuits in selected patient subgroups.

Overall, autoimmune-like signatures provide a useful framework for understanding how systemic adaptive immunity may intersect with CNS pathology in neurodegenerative dementias. The key question is not whether these disorders are autoimmune diseases, but whether defined immune signatures can reveal disease mechanisms, improve patient stratification, and identify rational windows for immune-informed intervention.
